# “We can’t pour water from an empty cup”: Caregivers’ Experiences with a Dementia Specific Training Program

**DOI:** 10.1093/geroni/igaf122.2593

**Published:** 2025-12-31

**Authors:** Brittney Pond, Kattia Suarez Vargas, Melinda Neri, Jarmin Yeh

**Affiliations:** Sandhills Community College, North Carolina, United States; University of California, San Francisco, San Francisco, California, United States; University of California, San Francisco, San Francisco, California, United States; University of California, San Francisco, San Francisco, California, United States

## Abstract

The demand for home and community-based services continues to rise in the United States, as does the prevalence of people living with Alzheimer’s Disease and Related Dementias (ADRD). However, little is known about the qualitative impacts of dementia specific training programs for California’s Medicaid-Funded In-Home Supportive Services (IHSS) caregivers who provide care and services for people living with ADRD. The IHSS + ADRD Training Project implemented and evaluated a 10-week, online, dementia-specific training program offered in English, Spanish, and Chinese (Cantonese and Mandarin) for IHSS caregivers and their care recipients living with ADRD. This poster is based on open-ended qualitative responses (n = 470) drawn from a larger quantitative survey of IHSS caregivers who participated in the training program. Caregivers responded to questions about what they learned in the program, its application to everyday work and life, personal impacts, and how the training could be improved. Based on our qualitative analysis using thematic coding, there are three main findings: 1) increased dementia knowledge and skills, 2) the importance of social support from classmates and instructors, and 3) personal development related to self-care and increased confidence. This research has several implications for policy and practice, as it provides evidence to support the ongoing development and implementation of dementia-specific caregiver training programs. This work suggests that establishing policies to support training opportunities are vital for this workforce.

